# Effects of Hypoxia on Cerebral Microvascular Angiogenesis: Benefits or Damages?

**DOI:** 10.14336/AD.2022.0902

**Published:** 2023-04-01

**Authors:** Yuying Guan, Jia Liu, Yakun Gu, Xunming Ji

**Affiliations:** ^1^Beijing Institute of Brain Disorders, Laboratory of Brain Disorders, Ministry of Science and Technology, Collaborative Innovation Center for Brain Disorders, Beijing Advanced Innovation Center for Big Data-based Precision Medicine, Capital Medical University, Beijing, China; ^2^Department of Neurosurgery, Xuanwu Hospital, Capital Medical University, Beijing, China

**Keywords:** angiogenesis, hypoxia, cerebrovascular microcirculation, endothelial cells, intermittent hypoxia

## Abstract

Cerebrovascular microcirculation is essential for maintaining the physiological functions of the brain. The brain can be protected from stress injury by remodeling the microcirculation network. Angiogenesis is a type of cerebral vascular remodeling. It is an effective approach to improve the blood flow of the cerebral microcirculation, which is necessary for preventing and treating various neurological disorders. Hypoxia is one of the most important regulators of angiogenesis, affecting the sprouting, proliferation, and maturation stages of angiogenesis. Moreover, hypoxia negatively affects cerebral vascular tissue by impairing the structural and functional integrity of the blood-brain barrier and vascular-nerve decoupling. Therefore, hypoxia has a dual effect on blood vessels and is affected by confounding factors including oxygen concentration, hypoxia duration, and hypoxia frequency and extent. Establishing an optimal model that promotes cerebral microvasculogenesis without causing vascular injury is essential. In this review, we first elaborate on the effects of hypoxia on blood vessels from two different perspectives: (1) the promotion of angiogenesis and (2) cerebral microcirculation damage. We further discuss the factors influencing the dual role of hypoxia and emphasize the benefits of moderate hypoxic irritation and its potential application as an easy, safe, and effective treatment for multiple nervous system disorders.

Normal cerebral microvasculature (arterioles, capillaries, and venules) is crucial for brain function [1, 2]. Microvasculature is required to maintain a sufficient blood supply for brain activity and the physiological functions of neurons and other cell types in the brain. To meet the demands of the brain, the microvasculature is involved in the feedback regulation of brain blood flow by participating in neurovascular coupling with neurons and cerebral autoregulation [1, 3, 4]. Microvessels are coupled with neurons to form the neurovascular unit (NVU). This reflects the close structural and functional relationship between nerve cells and the microvascular system as well as their cooperative effect in response to injury [5]. These are beneficial for nourishing the brain without functioning in energy storage and maintaining homeostasis in the central nervous system (CNS) [3]. Consequently, disorders of the microvasculature are associated with the occurrence and development of various CNS diseases [6]. For example, acute cerebrovascular embolism can cause stroke, and chronic cerebral microvascular pathology is a recognized cause of neurodegenerative diseases, including Alzheimer’s disease (AD) and Parkinson’s disease (PD) [7-10]. However, angiogenesis rarely occurs in healthy adults because the cerebrovascular structure is relatively stable. Furthermore, cerebrovascular remodeling gradually decreases with age, inhibiting an effective and timely response to protect the brain against stress-mediated damage [11, 12]. Increasing capillary density by inducing angiogenesis and improving microcirculation of the cerebral microvasculature can alleviate the negative effects of a stressful even on the brain [13, 14]. Thus, angiogenesis has the potential to play a key role in CNS disease prevention and control.

Hypoxia is a principal factor that induces angiogenesis in the cerebral vasculature. The process is accomplished by multiple angiogenic growth factors, including vascular endothelial growth factor-A (VEGF-A), angiopoietin-2 (ANGPT2), and erythropoietin (EPO), and is directly regulated by hypoxia-inducible factor-1α (HIF-1α) [15-20]. Although hypoxia stimulates angiogenesis, evidence suggests that cerebrovascular damage is caused by hypoxia resulting in increased permeability of the blood-brain barrier (BBB) and disjoined neurovascular coupling [21, 22]. Hypoxia has a dual effect, being both positive and negative for the structure and function of the cerebral microvasculature. These effects are mainly mediated by oxygen concentration and hypoxia duration and frequency. Therefore, it is important to review the different outcomes of the brain’s blood vessels as affected by various factors and to understand which factors contribute to improving the microcirculation of the brain. Promotion of favorable angiogenesis without causing brain damage would help prevent CNS disorders induced by hypoxia.

This review describes the processes and molecular mechanisms of microvascular angiogenesis in the brain. The effects of hypoxic signals on the different stages of microvascular angiogenesis were investigated in depth. We have also emphasized that severe hypoxia is harmful to the structure and function of the cerebral microvasculature. Finally, the main factors that cause the dual effect of hypoxia on cerebrovascular vessels (e.g., severity, duration, and mode) were analyzed to define suitable hypoxic parameters to improve the function of the cerebral microvasculature. This information will assist in the treatment and intervention of cerebral vessel-related diseases.

**Figure 1.**
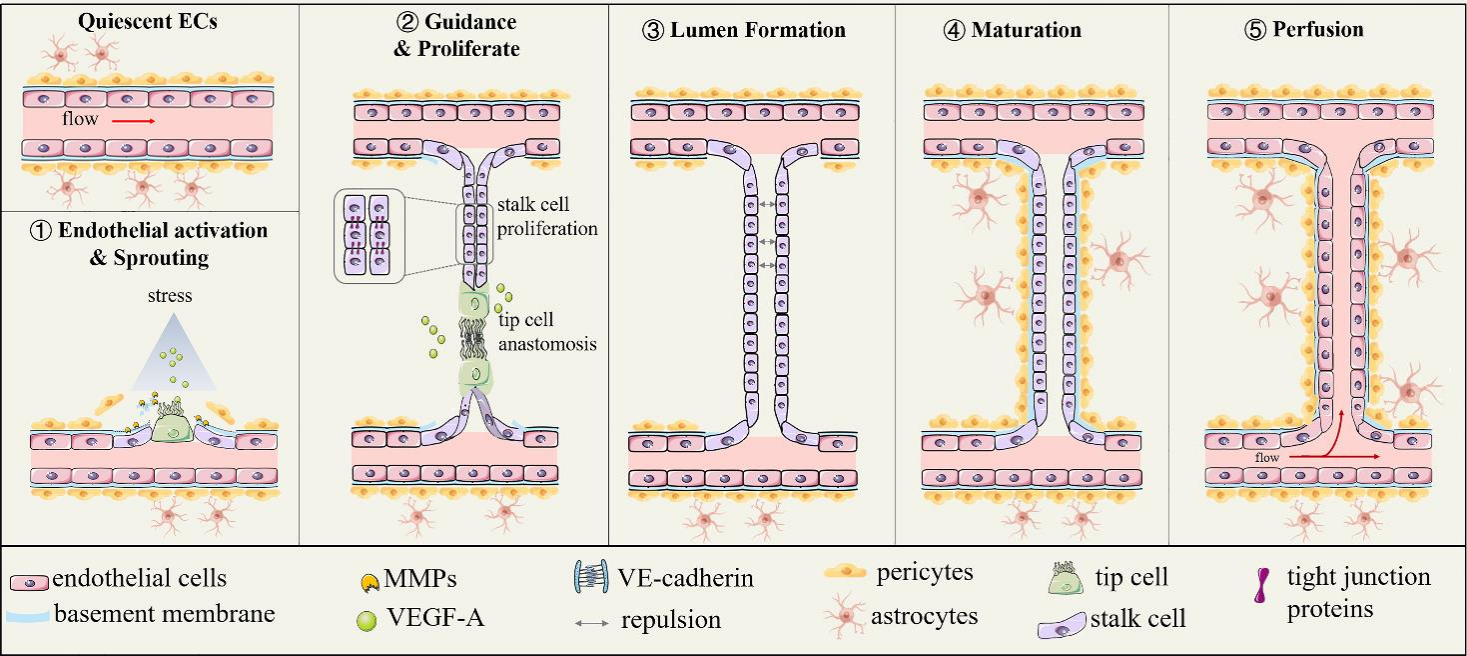
Stages of angiogenesis. (A) Quiescent endothelial cells (ECs): mature capillaries consist of quiescent ECs surrounded by mural cells and basement membranes. (B) Endothelial activation: in response to hypoxia stimulation, vascular endothelial growth factor-A (VEGF-A) activates matrix metalloproteinases (MMPs), and other factors are increased and bind to corresponding receptors, resulting in the degradation of the basement membrane and detachment of pericytes and polarization of ECs. (C) Sprouting: tip cells and stalk cells are selectively differentiated under the stimulation of different angiogenic factors. (D) Guidance and lumen formation: (1) tip cells navigate following the VEGF-A concentration gradient while stalk cells elongate and proliferate; (2) vascular endothelial cadherin facilitates tip cell anastomosis between branches; and (3) lumen formation is due to intercellular polarity rejection. (E) Vessel maturation and perfusion: (1) mural cells in the vessels are recruited and the basement membrane reforms. (2) Upon perfusion, active stalk cells dynamically change shape and oxygen reduces VEGF-A expression shifting endothelial behavior toward a quiescent phenotype.

## 1. Cerebral microvascular angiogenesis

Cerebral vascular remodeling can increase cerebral blood flow by improving cerebral vascular microcirculation and has curative and lasting protective effects on the brain to relieve stress-related injuries. There are two main types of cerebrovascular responses: arteriogenesis and angiogenesis. Arteriogenesis is defined as short-term dilation of blood vessels to improve blood flow. Angiogenesis is defined as the formation of new microvessels from the pre-existing vasculature [23]. Angiogenesis has a more continuous effect than arteriogenesis. This review focuses on the processes and mechanisms of angiogenesis and the effects of hypoxia on angiogenesis. At present, angiogenesis is believed to consist of three stages: sprouting, proliferation, and maturation [24, 25] as described below and shown in Figure-1.

### 1.1 Sprouting stage of angiogenesis

Vascular sprouting is the first important step in angiogenesis and is polarized mainly into tip cells from parts of the endothelial cells (ECs). Tip cells are specialized cells located at the forefront of the growing blood vessels [26]. They are regulated by hypoxic stress and signaling through various vascular growth factors around the vessels that activate their migration ability [23]. The adhesion of pericytes physiologically inhibits the polarization of tip cells; therefore, angiogenesis does not occur [23]. However, hypoxia is a crucial regulator that induces angiogenesis and transforms normal brain microvascular ECs (BMECs) into polarized tip cells [23]. HIF-1α is specifically stable under hypoxia and mediates a series of reactions. In the absence of oxygen, HIF-1α directly activates the transcription of angiogenic factors, such as VEGF-A and ANGPT2 [26, 27]. These proteins induce the detachment of vascular cells from the basement membrane (BM), activate matrix metalloproteinases (MMPs) to degrade the extracellular matrix, and guide tip cells to sprout in a particular direction [28-30]. The concentration gradient of VEGF-A is a major inducing factor for tip cell migration [31], that binds to corresponding receptors to guide tip cell growth in a fixed direction. VEGFR1 and VEGFR2 are receptors of VEGF-A, but they play different roles. Overall, the binding of VEGFR2 and VEGF-A positively promotes tip cell migration, whereas VEGFR1 negatively regulates this process [32]. The soluble VEGFR1 secreted by ECs inactivates VEGF-A around the emerging sprout to maintain proper direction for tip cells [33]. VEGFR1 on EC membranes inhibits angiogenesis via VEGFR2 signaling to avoid vascular growth in an uncontrolled manner [34]. Moreover, neuronal VEGFR2 binds to and consumed VEGF-A around that would otherwise contribute to tip cells toward neurons incorrectly [35].

### 1.2 Proliferation stage of angiogenesis

After sprouting, the BMECs proliferate for a while, forming tubules based on the rule of directional arrangement. This phenomenon is called lumen formation during angiogenesis. Without tubulogenesis, the endothelium fails to transport fluids and the essential components of the blood [29]. The direction of tubule movement is regulated by angiogenic signals. Encounters between two sprouts can contribute to anastomosis and tube formation at a specific location and establish an angiogenesis network, which is always driven by hypoxia [23]. As tip cells anastomose with cells from the surrounding sprouts, stalk cells proliferate to form new vessels and branches [29, 31, 36]. As blood vessels proliferate to form tubules or eventually to form a network of vessels, the tip cells are responsible for receiving the signals that guide the direction and where they meet [37]. Stalk cells, another type of proliferative endothelial cells, line up to allow the nascent sprout to proliferate and elongate [29, 31, 36, 38]. Various downstream molecules of hypoxia signaling, such as VEGF and ANGPT, are also involved in this process; these molecules coordinate with each other or antagonize regulation to ensure the correct and orderly arrangement of BMECs to form functional vessels [15, 38]. Among them, VEGF-A, vascular endothelial cadherin (VE-cadherin), ANGPT (ANGPT1 and ANGPT2), and other pro-angiogenesis factors play a key role [15-20]. ANGPT plays both proangiogenic and angiogenic roles [37, 38]. In addition, the expression of tight junction proteins, including claudin-5 and zonula occludens-1 (ZO-1), ensure vascular endothelial migration, tube formation, and tight connections [39-41].

### 1.3 Maturation stage of angiogenesis

Vascular maturation is an important step for new blood vessels to become functional with complete structure and function. This process primarily involves the formation of a complete BBB. During angiogenesis, after ECs have completed their arrangement and tube formation during the vascular proliferation stage, ECs further interact with other cells during the vascular maturation stage, thus forming the BBB. The BBB is a structure unique to the brain that guarantees selective transport of peripheral and central substances, thereby maintaining homeostasis in the brain [42]. The BBB mainly includes basal membranes, vascular ECs, tight junction proteins between them, pericyte cells, and the feet of astrocytes [43, 44]. ECs secrete platelet-derived growth factor-β (PDGF-β), angiopoietin-1 (ANGPT1), transforming growth factor-β (TGF-β), and other factors to recruit pericyte cells and astrocytes, which cover the surface of ECs. The inhibition of MMPs by metalloproteinase tissue inhibitors (TIMPs) and plasminogen activator inhibitors-1 (PaI-1) prompts the re-formation of the basement membrane [45]. With the infusion of blood, the hemodynamic signals of the local vasculature are used to trim and modify the vascular network to establish an effective perfusion vascular bed to meet the oxygenation needs of the tissue [46]. Besides, the delivery of oxygen and nutrients reduces VEGF expression and inactivates polarized ECs to a quiescent phenotype [40]. Together, these processes promote the formation of a functional BBB via angiogenesis, which transforms new branches into mature microvasculature. This contributes to the regulation of blood flow and neurovascular coupling as well as the interactions with neurons and other cells, such that the NVU is formed. The mature NVU not only transports O_2_ and glucose through the bloodstream, but also participates in physiological processes, such as protein homeostasis, neuro-immune regulation, waste removal, and brain temperature regulation [47, 48]. Therefore, vascular maturation is necessary for the formation of functional blood vessels. Other cells contributing toward a blood vessel disorder also lead to the destruction of the BBB and vascular leakage, resulting in secondary nerve damage [49].

Thus, angiogenesis is a physiological response in which hypoxia induces the spatiotemporal expression of various angiogenic factors and their interaction with cells. In the following sections, we have reviewed the role of hypoxia in different stages of angiogenesis.

## 2. Effects of hypoxia on cerebral microvascular angiogenesis

Cerebrovascular remodeling regulates blood flow to meet the oxygen and nutrient requirements of brain tissue. Angiogenesis is one of the main mechanisms involved in cerebral vascular remodeling and the improvement of cerebral blood flow [23]. Hypoxia signals activate and regulate multiple steps in cerebral angiogenesis, suggesting that the hypoxic response plays an important role in angiogenesis.

The next topic will focus on explanations of hypoxia and intracellular response mechanisms to hypoxia. The role and molecular mechanisms of hypoxia signals in the different stages of cerebral microvascular angiogenesis are discussed in depth.

### 2.1 Hypoxia and the response to hypoxia

Oxygen is a key molecule that provides energy to the body, tissues, and cells and maintains physiological functions [50]. Hypoxia occurs when the ambient oxygen concentration falls below the normal oxygen concentration in the air (typically 21%), or when oxygen consumption by an organism exceeds the oxygen supply [51, 52]. Hypoxia caused by the external environment occurs during environmental changes, such as altitude exposure, while tissue hypoxia in the body occurs in senescence, disease, and other states [52-54]. A series of response mechanisms are activated in bodies exposed to hypoxia, including increased ventilation and erythrocyte volume. In cells, the response to hypoxia is mainly mediated by hypoxia-inducible factors (HIFs) [55, 56]. After activation, HIFs upregulate a series of hypoxia response factors that play a defensive role [57]. The HIF family consists of three members, HIF-1, HIF-2, and HIF-3. In the presence of sufficient oxygen, the factor inhibiting HIF (FIH) prevents the activation of HIF-α proteins, and prolyl-hydroxylases (PHDs) induce the degradation of HIF-α proteins. However, both FIH and PHDs are inactive under hypoxic conditions, resulting in HIF-α protein stabilization [55]. Subsequently, stable HIF-α enters the nucleus and binds to HIF-β, thereby stimulating the transcription of a series of downstream molecules, including various molecules associated with angiogenesis, such as VEGF-A, ANGPT2, and EPO [15-20]. Therefore, the highly ordered, stable, and functional new vessels formed by angiogenesis depend on the coordination of cellular and extracellular signaling molecules and morphogenic signals [38].

Next, we will explore the role of hypoxia in cerebral angiogenesis and the regulatory mechanisms of these above key molecules in this process.

### 2.2 Hypoxic conditioning

Although the body initiates a series of mechanisms in response to hypoxia, the protective effect generated is insufficient to prevent injury. Hypoxia adaptation is described as exposure to moderate hypoxia with the intention of stimulating the body’s endogenous protective mechanism and increasing the body’s tolerance to hypoxia to resist more serious hypoxia or ischemic injury. Common hypoxia adaptation methods include hypoxic preconditioning and intermittent hypoxic treatment, which have been widely studied in recent years as potential non-drug therapies for many diseases, including CNS diseases. Adaptation to hypoxia is considered crucial to improve cerebrovascular microcirculation and provide protection against stress [58, 59]. However, at present, the effects of hypoxia adaptation on the cerebrovascular system remain inconclusive. While hypoxia adaptation can promote angiogenesis and improve cerebrovascular microcirculation, studies have shown that hypoxia preconditioning is ineffective and even causes injury [21, 22].

In the following paragraphs, we discuss the influence and mechanism of hypoxia adaptation intervention on the cerebrovascular system and elaborate on its role and curative effect in CNS diseases.

### 2.3 Beneficial effects of hypoxia on cerebral microvascular angiogenesis

VEGF-A is a multipotent cytokine involved in many physiological and pathological processes, including direct stimulation of angiogenesis. VEGF-A is the strongest activator of angiogenesis in CNS [60]. In the sprouting stage of angiogenesis, HIF-1α activation promotes VEGF-A transcription. VEGF-A specifically binds to phosphorylated VEGFR2 and induces BBB degradation before the arrival of new vessels. It splits vascular mural cells and basement membranes and degrades those basement membranes and endothelial tight junction proteins [61]. BMECs without wrapping are further polarized as tip cells by VEGF-A to initiate angiogenesis [41, 62]. In addition, VEGF-A promotes stalk cell proliferation during the extension of new vessels to support alignment and tubulogenesis [63]. Therefore, VEGF-A plays an indispensable role in the selection of tip cells and induction of stalk cells in adjacent cells, as well as the migration, proliferation, and formation of new functional vascular branches. This has been verified in various animal models. In addition, VEGF-A expression is related to mural cell recruitment and pruning of unstable vessels [64]. Atmospheric hypoxia upregulates the expression of HIF-1-dependent EPO and VEGF-A in the brain to enhance tolerance to focal permanent cerebral ischemia [41, 65]. Long-term hypoxia exposure significantly increases brain capillary density [66].

The ANGPT/Tie pathway plays an important role in regulating vascular stability, angiogenesis under physiological and pathological conditions, and inflammation [67]. The ANGPT/Tie pathway comprises two type-I tyrosine kinase receptors (Tie1 and Tie2) and four ligands (ANGPT1, ANGPT2, ANGPT3, and ANGPT4) [68-70]. ANGPT1 principally maintains the stability of vascular ECs and the interaction between ECs and other cells, whereas ANGPT2 participates mainly in the formation of vessels. However, the characteristics of ANGPT3 and ANGPT4 are poorly understood [71]. Angiogenesis mediated by ANGPT1 and ANGPT2 is largely dependent on their interaction with Tie2. ANGPT1 physiologically binds to and phosphorylates the Tie2 receptor, resulting in downstream signaling that promotes cell survival and vascular stability [68, 71]. The activation of HIF-1α in BMECs exposed to stress induces the upregulation of ANGPT2. High levels of ANGPT2 compete with ANGPT1 to cause shedding of BBB-related cells and instability of BMECs [40, 72]. This renders vessels sensitive to the effect of VEGF-A, stimulating the sprouting of BMECs [68, 73]. During the proliferation of new vessels, ANGPT2 expression is low and Tie2 expression is high in stalk cells, which is conducive to transmitting ANGPT1/Tie2 signaling, leading to vascular remodeling and stabilization [37]. During vessel maturation, ANGPT1 activates Tie2 in pericytes, promotes BMECs survival, inhibits further proliferation of BMECs, and stabilizes the binding between pericytes and BMECs, which promotes the maturation of newly formed vessels [40]. In addition, independent of Tie2, ANGPT2 promotes angiogenesis in other pathways, mainly by binding to integrin [74]. ANGPT2 interacts with β1 integrin through phosphorylated FAK and activates Rac1 to induce angiogenesis sprouting and cell migration. This pathway is Tie2-independent [37]. Normobaric hypoxia (8% O_2_ for 24 h) increases the expression of ANGPT2 in BMECs and pericytes of cerebral blood vessels in mice [75]. Such hypoxic treatment for 2 weeks also upregulates the fibronectin-integrin α5β1 axis in BMECs, so that BMECs proliferate, and angiogenesis increases cerebral vascular density by more than 50% in all brain regions of rodents [76, 77].

EPO is a pleiotropic factor that stimulates the growth and differentiation of many cells and tissues by binding to the homodimeric EPO receptor (EPOR), heterodimeric receptor (EPOR), and common β receptor (βC-R) [78]. EPO and its receptors are expressed in various cells of the nervous system including neurons, astrocytes, and ECs [79-81]. The EPO/EPOR pathway is required for normal brain functions [82]. It has been proven to play a protective role under various stress conditions (such as ischemia and hypoxia) in the CNS by promoting angiogenesis, preventing apoptosis, and reducing inflammation [79, 83]. EPO directly binds to its receptors to activate angiogenesis pathways, and promotes angiogenesis by indirectly increasing VEGF levels. The direct binding of βC-R and EPO signaling activates endothelial nitric oxide synthase [84], which plays a critical role in vascular growth [85]. The direct binding of EPO and heterodimeric EPOR/βC-R activates endothelial progenitor cells, which is the key process involving βC-R interacting with VEGFR2 in the pathway [78]. In addition, Wang et al. reported that the vascular regulatory pathway of EPO is related to VEGF [86]. Administration of rhEPO increased cerebral VEGF levels and EPO-induced capillary-like tube formation was blocked by a specific VEGF receptor 2 antagonist, suggesting that VEGF mediates EPO-enhanced angiogenesis [86]. However, Liu et al. reported that EPO increases VE-cadherin expression to maintain vascular integrity and barrier function in experimental retinopathy by inhibiting the VEGF/VEGFR2/Src signaling pathway [87]. In hypoxia, the activation of HIF-2α promotes EPO gene transcription and upregulates EPO expression, which is involved in the migration and proliferation of vascular ECs [80, 88]. Wang et al. reported that recombinant EPO (rhEPO) significantly improved functional recovery and increased the density of cerebral microvessels and neurogenesis 24 h after experimental hypoxic-ischemic injury in rats [86]. Li et al. confirmed that intra-abdominal administration of rhEPO after focal cerebral ischemia could reduce cell death of BMECs, enhance the outcomes of angiogenesis, and significantly restore local cerebral blood flow [89]. Furthermore, the administration of rhEPO plays a protective role in acute cerebral focal ischemia, mainly by reducing BBB leakage and cerebral edema [90]. In addition, hypoxia adaptation to 8-11% O_2_ before middle cerebral artery occlusion (MCAO) in mice could upregulate the expression levels of growth factors, such as EPO and VEGF-A, and reduce to some extent the volume of cerebral infarction [41, 91-93].

**Table 1:** Effects of consistent hypoxia (CH) on cerebral angiogenesis.

Consistent mild hypoxia (12% ≤ FIO2 < 21%)						
FIO2	Duration	Subjects	Effects			Refs
			Changes in angiogenic factors	Positive effects of angiogenesis	Negative effects on CNS	
16%	7 d	tMCAO mice	claudin-5 without change	MVD without change	Not observed	[110]
14%	7 d	tMCAO mice	claudin-5↑,α5 integrin↑	MVD without change	Not observed	[110]
13%	7 d	tMCAO mice	claudin-5↑,α5 integrin↑	BMECs↑,the area of microvessel ↑but the area of large vascular and whole brain vessels without change	Not observed	[110]
12%	7 d-14 d	C57BL/6 mice,pMCAO mice	claudin-5↑, α5 integrin↑	microvessel area↑, MVD↑, infarct volume after reoxygenation for 6w↓	Not observed	[110, 140]
Consistent moderate hypoxia (8% ≤ FIO2 < 12%)						
FIO2	Duration	Subjects	Effects			Refs
			Changes in angiogenic factors	Positive effects of angiogenesis	Negative effects on CNS	
11%	2-6 h	tMCAO mice	EPO↑, VEGF-A↑	infarct volume↓	N	[41, 91]
10%	24 h	C57BL/6 mice	VEGF-A↑	N	BBB leakage	[97]
	7 d	MCAO mice, Wistar rats	claudin-5↑, α5 integrin↑	microvessel area↑, MVD↑, regional blood flow↑	N	[110, 140, 141]
	2 w	EAE mice	ZO-1↑, occludin↑	N	N	[142]
	3 w-8 w	C57BL/6 mice,pMCAO Wistar rats	N	microvessel area↑, infarct volume↓	N	[140, 143, 144]
8%-8.5%	6 h	MCAO mice	EPO↑	infarct volume↓	brain edema↑	[68, 99]
	24-48 h	C57BL/6 mice	VEGF-A↑	N	BBB leakage	[97, 105]
	2-3 w	C57BL/6 mice, MCAO mice, Wistar rats	claudin-5↑, α5 integrin↑, laminin↑	microvessel area↑, MVD↑	capillary leakage	[76, 77, 98, 110, 140, 141, 144-147]
Consistent severe hypoxia (FIO2 < 8%)						
FIO2	Duration	Subjects	Effects			Refs
			Changes in angiogenic factors	Positive effects of angiogenesis	Negative effects of CNS	
7%	6 h	Kunming mice	N	N	neurons died, BBB leakage	[104]
	14 d, after 14 days of hypoxia adaptation	tMCAO mice	N	MVD↑	N	[140]
6%	1 h, 24 h	C57BL/6 mice; rats	VEGF-A↑	N	BBB leakage	[97, 104]

Annotation: AD, Alzheimer’s disease; BBB, blood-brain barrier; EAE, experimental allergic encephalomyelitis; EPO, erythropoietin; MVD, microvessel density; N, not mentioned; pMCAO, permanent middle cerebral artery occlusion; SO_2_, O_2_ saturation; tMCAO, transient middle cerebral artery occlusion; VEGF-A, vascular endothelial growth factor-A; ZO-1, zonula occludens-1.

### 2.4 The damage effects of hypoxia on cerebral microvascular angiogenesis

Although hypoxia promotes angiogenesis through these pathways, many studies have shown that hypoxia is the main stressor in the destruction of the BBB [94-97]. A recent study by Sebok et al. demonstrated that hypoxia-induced leaky cerebral vasculature did not colocalize significantly with angiogenesis labeled with bromodeoxyuridine (BrdU), suggesting that hypoxia-induced vascular damage may be extensive and not due to new blood vessels failing to mature [98]. In experiments using an animal model of ischemia and hypoxia, Mellender et al. showed that hypoxic pretreatment (8% O_2_ for 2-48 h) increased BBB permeability through VEGF-related pathways and exacerbated cerebral edema in a permanent MCAO rat model [99]. Gomes et al. reported that in a chronic intermittent hypoxia (CIH) model of 3-5 weeks, alternating between 5% O_2_ and 21% O_2_ for 8 h caused vascular damage [100]. Hypoxic treatment at an altitude of 5 km (nearly 13% O_2_) for 30 min followed by an altitude of 8 km (nearly 8% O_2_) for 48 h reduced collagen and elastic fiber content in the cerebrovascular system of mice, resulting in decreased cerebrovascular function [101]. During hypoxic intervention alone, after hypoxia (6% O_2_ for 1 h followed by 20 min normoxia), the BBB of female Sprague-Dawley rats showed increased permeability to small sucrose molecules, but the permeability to Evans blue reabsorbed by large protein molecules did not change [102, 103]. This suggested that acute hypoxia affected the BBB permeability to small molecules rather than macromolecules. Zhao et al. found that prolonged hypoxia (7% O_2_ for 6 h) increased BBB permeability to Evans blue dye in mice [104]. In addition, Schoch et al. and Bauer et al. successively proved that hypoxia (8% O_2_ for 24 or 48 h) increased the leakage of fluorescein sodium from the BBB in mice, which was associated with a significant decrease in occludin expression and changes in occludin localization [97, 105].

## 3. Hypoxia parameters affecting the effects on cerebral microvascular angiogenesis

**Figure 2.**
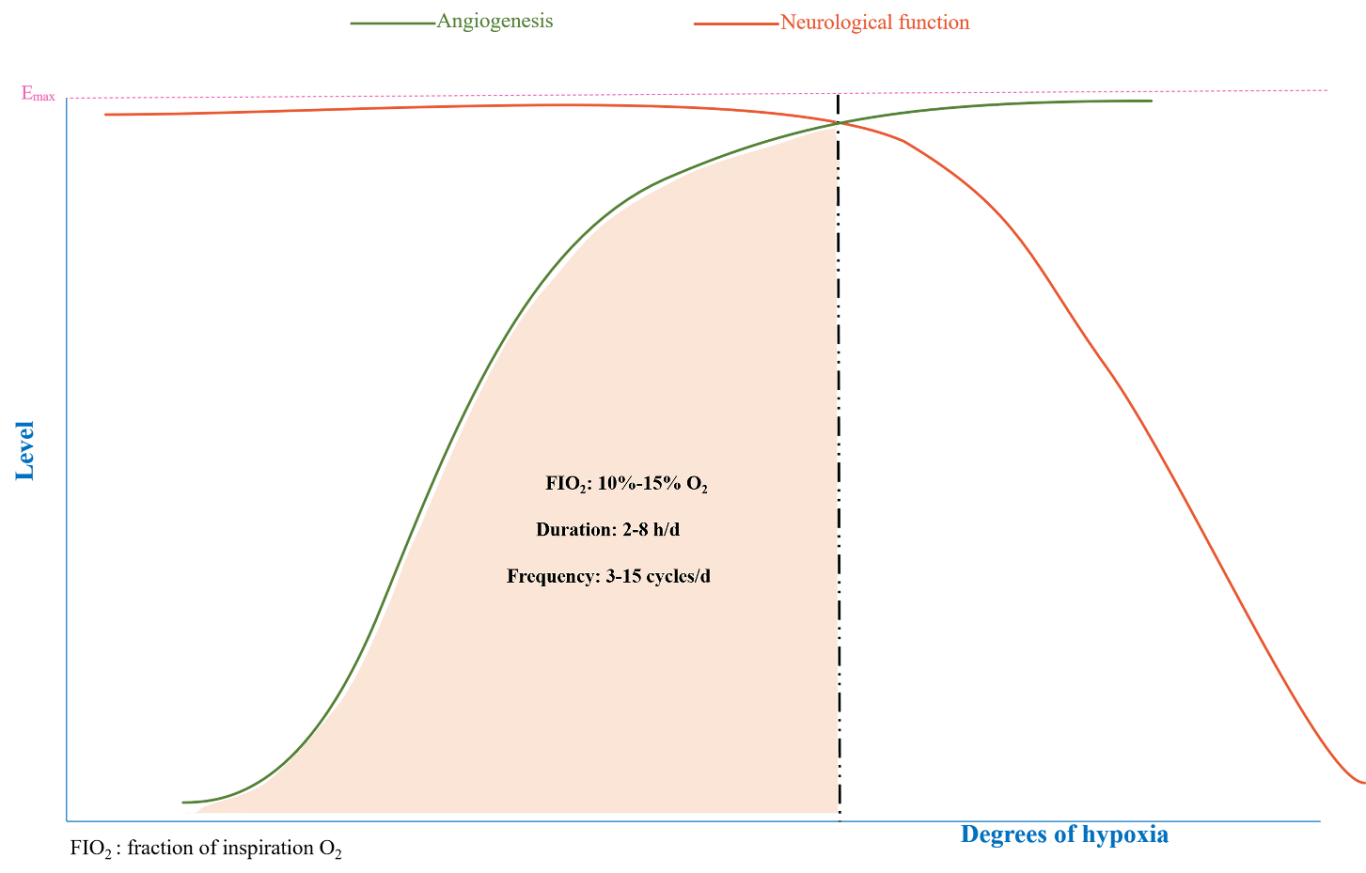
Schematic of the role of hypoxia. The balance of critical factors determines beneficial vs. pathogenic roles of hypoxia on the cerebral microvasculature. The hypoxic intervention (5-8% O_2_, more than 50 cycles/day or more than 8 h/day) is prone to neurological damage. Quite mild hypoxia (15-21% O_2_; less than 2 h/day) does not stimulate angiogenesis. In contrast, moderate hypoxia (10-15% O_2_; 2~8 h/day; <15 cycles/day) seems to contribute to beneficial (potentially “therapeutic”) effects with minimal nerve damage (shaded in blue).

The dual effects of hypoxia on angiogenesis have been described previously. Although hypoxia promotes angiogenesis of cerebral microvessels and the growth of vascular branches, hypoxia also causes cerebral microvascular injury. However, the two outcomes of hypoxia are largely determined by a number of key determinants, including oxygen concentration, hypoxia duration, hypoxia frequency, and hypoxia patterns, which are discussed below and summarized in Tables 1 and 2 and Figure 2.

### 3.1 The oxygen concentration

The degree of hypoxia depends mainly on reduced oxygen concentration. It is often difficult to stimulate the tolerance mechanism of the body when the hypoxia level is mild, while an extremely severe hypoxia level is not only harmful to the CNS but is also an important factor in the development of other systemic diseases. In animal models, reducing arterial O_2_ partial pressure (PaO_2_) to 45-50 mmHg (moderate hypoxia exposure) has been shown to have a protective effect [106]. Similarly, in humans, 45-50 mmHg of PaO_2_, corresponding to an arterial SO_2_ of 70-75% and a brain SO_2_ of 50-55%, is protective [107, 108]. Such moderate hypoxic exposure results in vasodilation, which delivers sufficient O_2_ to meet the metabolic needs of the brain [108]. Severe hypoxic conditions, such as 8% O_2_, lead to severe hypoxemia [109]. While 8% O_2_ stimulates intense remodeling of blood vessels in the brain, it also triggers severe damage to the blood vessels of the brain [98]. Therefore, determining the appropriate hypoxic concentration is the basis for identifying the best hypoxic option and exploring the efficacy of hypoxic-related interventions in diseases.

Boroujerdi et al. maintained wild-type C57BL/6 mice aged 8-10 weeks under hypoxia for 7 days at different oxygen levels of 8%, 10%, 12%, 14%, and 16% O_2_. When the ambient O_2_ levels were higher than or equal to 14%, it was difficult to induce an extensive increase in the number of vessels. However, levels lower than 14% can induce significant cerebral angiogenesis and remodeling. Therefore, the hypoxia threshold level for stimulating cerebrovascular remodeling is between 12% and 14% O_2_ [110]. Further studies have shown that 13% O_2_ induces cerebral microangiogenesis by promoting BMEC proliferation without significant macrovascular dilatation. Therefore, 13% O_2_ may be a safe and effective threshold oxygen concentration for inducing cerebral microangiogenesis [110].

**Table 2:** Effects of intermittent hypoxia (IH) on cerebral angiogenesis.

Intermittent mild hypoxia (12% ≤ FIO2 < 21%)									
FIO2	Duration per day	Single intervals	Frequency	Total course	Subjects	Effects			Refs
						Changes in angiogenic factors	Positive effects of angiogenesis	Negative effects of CNS	
14.5%	10 min	-	1	6 w, 3 d/w	healthy human during exercise	VEGF-A↑	N	N	[148]
13%	9 h	2 min	135	2 w	healthy human	N	N	Blood Pressure↑	[149]
12%	50 min	6 min hypoxia-4 min normoxia	5	3 d/w	healthy human	N	MVD↑, BMCE function↑	N	[150]
	30 min	4 min	3	1 d	healthy human	EPO without change	ischemia reperfusion injury↓	N	[56, 122]
12%-10%	70 min	5 min	7	4 w,5 d/w	healthy human	EPO and SO2 without change	N	N	[123, 124]
Intermittent moderate hypoxia (8% ≤ FIO2 < 12%)									
FIO2	Duration per day	Single intervals	Frequency	Total course	Subjects	Effects			Refs
						Changes in angiogenic factors	Positive effects of angiogenesis	Negative effects of CNS	
8%-11%	2-4 h	-	1	2 w	tMCAOmice	EPO↑;VEGF-A↑	infarct volume after hypoxia 8w↓	BBB permeability↑	[92, 93]
10-11%	12 h	90 s	240	2 d	Sprague Dawley rats	N	N	learning ability↓; cognitive function↓	[125]
10%	8 h	90 s	160	35 d	tMCAOmice	EPO↑,VEGF-A↑	infarct volume↓	N	[151]
	6-8 h	2 min	90~120	4 d	healthy human	N	N	cerebral vascular resistance↑, cerebral autoregulatory↓	[126]
	2 h	6 min hypoxia-4 min normoxia	12	21 d	AD mice	EPO↑	N	N	
Intermittent moderate hypoxia (FIO2 < 8%)									
FIO2	Duration per day	Single intervals	Frequency	Total course	Subjects	Effects			Refs
						Changes in angiogenic factors	Positive effects of angiogenesis	Negative effects of CNS	
6%	8 h	90 s	160	35 d	tMCAOmice	EPO↑	N	infarct volume↑	[151]
5%	8 h	~10 min	24-160	3-5 w	C57Bl/6mice, AD mice	N	N	cognitive function↓, MVD↓	[100, 152]

Annotation: AD, Alzheimer’s disease; BBB, blood-brain barrier; EPO, erythropoietin; MVD, microvessel density; N, not mentioned; SO_2_, O_2_ saturation; tMCAO, transient middle cerebral artery occlusion; VEGF-A, vascular endothelial growth factor-A.

### 3.2 The duration of hypoxia

In addition to oxygen concentration, the duration of hypoxia also affects the blood vessels differently. Generally, the hypoxia duration can be divided into consistent hypoxia (CH) and intermittent hypoxia (IH). Although CH can promote angiogenesis, it often induces vascular and nerve damages. IH is a safer and more effective treatment strategy than CH. IH preconditioning is commonly used to train pilots, mountain climbers, and athletes to improve their operational ability and performance by adapting to a hypoxic environment [111-113]. In recent years, an increasing number of studies have proven that IH preconditioning is beneficial for treating chronic cardiopulmonary diseases, iron-deficiency anemia, ischemic coronary heart disease, neuro-degenerative diseases, and other vascular-related diseases [114-117]. Mechanistically, the promotion of angiogenesis is an important pathway through which IH plays a protective role [118-120]. However, whether IH is realistically safe and effective also varies depending on the length of each intermission, meaning that too short a stimulation fails to achieve the desired effect, while too long a stimulation causes additional damage to the body [121]. Studies have shown that IH (10-12% O_2_ for 30 or 70 min per day) in healthy adults does not cause significant changes in angiogenesis-related factors, such as EPO and VEGF-A [56, 122-124]. Ackman et al. demonstrated that a single exposure duration of less than 2 h at 10% O_2_ did not cause significant changes in pro-angiogenic factors (such as EPO), while a duration longer than 8 h resulted in decreased cerebrovascular function and impaired neurological function [125, 126]. Miller et al. reported that the expression of VEGF-A and EPO could be increased under 11% hypoxia for 2 to 6 hours, and played a protective role in the MCAO model [41, 91]. Therefore, a single exposure of 2 to 8 hours might provide a more protective outcome in terms of cerebrovascular angiogenesis. Further studies are required to confirm this hypothesis.

The duration of each hypoxic event and the total exposure time to hypoxia were also factors impacting the effect of IH. A single exposure to IH (12% O_2_ with dive cycles of 4-min intervals per day) was not sufficient to cause a significant increase in erythropoietin or hemoglobin content in young healthy individuals [56]. This suggests that IH intervention should be repeated many times to achieve better efficacy. However, this study was inconclusive regarding whether the total IH exposure time of IH is related to the curative effect. Therefore, further systematic studies are required.

### 3.3 The hypoxia model

As described previously, oxygen concentration, duration, and frequency of hypoxia have an important influence on the curative effect of hypoxia. Therefore, an optimal combination of the above factors may be the best mode for IH to play a protective role and avoid neurovascular injury. Studies have shown that 3-15 cycles per day are safe and effective IH patterns at oxygen concentrations of 9-16%. Higher IH frequencies (greater than 50 cycles per day) can induce sleep apnea syndrome-like symptoms and more severe hypoxemia [127]. However, so far, research on the best model remains limited. An in-depth study is crucial for promoting the clinical application of IH.

## 4. Limitations and conclusions

Here, we review relevant studies on the mechanism by which hypoxia preconditioning promotes cerebral angiogenesis and its effect on cerebrovascular diseases. Hypoxia can have both favorable and unfavorable consequences for the cerebrovascular system. While it promotes angiogenesis, it can result in damage. One approach is to characterize hypoxic parameters associated with safety, protection, and/or therapeutic effects and pathogenesis.

In this review, we found that compared with CH, IH intervention has a significant advantage, whereby severe injury may be avoided if a similar effect of angiogenesis is achieved. However, the effect of IH is influenced by a number of factors, including the oxygen concentration level, cumulative duration of hypoxia episodes, and hypoxia pattern (e.g., single cycle, single exposure time). Not surprisingly, severe IH regimens tend to be pathogenic, whereas any beneficial effects are more likely to result from moderate IH exposure. Moderate hypoxia (12-15% O_2_) and moderate duration (2-8 h per day) most often lead to pathological angiogenic effects, whereas severe hypoxia (< 8% O_2_) and a longer duration (> 8 h per day) gradually lead to aggravated pathological harm, including vascular injury. In conclusion, moderate IH may be an effective strategy for preserving the positive effects of hypoxia on cerebrovascular activity and reducing its damaging effects.

At present, studies on angiogenesis induced by both CH and IH still focus on angiogenesis itself but lack evaluation of the structural and functional integrity of angiogenesis, which hinders the clinical application of this therapy in the future. The structural integrity of angiogenesis depends primarily on the BBB formation. However, it is worth noting that the BBB has been studied largely as a static structure, which is a barrier that protects the CNS from potential toxins. The BBB is not a single structural unit, and only interacts with neurons and other nerve cells to dynamically respond to meet the constantly fluctuating metabolic needs of brain activity [128]. The close association between neurons and vascular function is one of the unique characteristics of cerebral circulation, and many studies have found that the two regulate each other. Neuronal activity is critical for local blood flow and angiogenesis [129, 130]. BBB can significantly alter the extracellular environment, thus regulating the function of the neural circuit network [131, 132]. Angiogenesis regulates the balance between proliferation and differentiation of neural progenitor cells (NPC). For example, hypoxic neurogenic niches enhance NPC proliferation, whereas increased blood supply through angiogenesis induces NPC differentiation [133]. Therefore, to study the role of hypoxia in inducing angiogenesis and improving cerebrovascular microcirculation in the future, more attention should be paid to the integrity of the BBB in angiogenesis and its interaction with other nerve cells. This will ensure that angiogenesis induced by the selected hypoxic mode is functional, which will contribute to future clinical transformation.

Furthermore, to facilitate the translation of basic to clinical research, it would be worth exploring whether the results of IH are affected by age, sex, brain region, and disease, and whether specific biomarkers could be characterized. Although the clinical application of hypoxia in cerebrovascular intervention is not yet extensive, it has been shown to be safe and effective for other diseases. IH conditioning (eight sessions of 14% O_2_ with an episode of 24-min interval, for 6 weeks) improves blood pressure, nitric oxide, and hypoxia-inducible factor-1 alpha in hypertensive patients [134]. Similarly, it is a potential utility of IH for preventing the development of type 2 diabetes. IH training (12% O_2_ with 4 cycles of 5-min intervals, for 3 weeks, 3 sessions per week) significantly reduced fasting glucose, increased tolerance to acute hypoxia, and stimulated gene expression in prediabetes patients [135]. Some studies have also suggested that IH exerts anti-inflammatory effects to protect tissues. Pro-inflammatory mediators of adult men exposed to IH (10% O_2_ with a 5-min interval for 14 days) were suppressed significantly, such as TNF-α and IL-4 [136]. Likewise, exposure to IH (12% O_2_ for 6 days/4 h per day) increased the levels of the anti-inflammatory interleukin IL-10 [137]. In addition, intermittent hypoxic-hyperoxic training, involving episodes of hypoxia alternating with moderate oxygen enrichment, has gradually become clinical practice. Bayer et.al. reported that 5-7 weeks of 2-3 days per week exposure to 4-8 hypoxia-hyperoxia cycles each session (4-7 minutes of 10 - 14% O_2_ and 2 - 4 minutes of 30 - 40% O_2_) improved cognitive function by 16.7% and exercise tolerance in geriatric patients [138]. In addition,15 sessions of intermittent hypoxic-hyperoxic training showed improved exercise capacity, reduced blood pressure, and enhanced left ventricular ejection fraction in patients with coronary artery disease [139]. Therefore, exploring the optimal mode of IH in greater depth is an important and potential angiogenesis approach to promote hypoxic-related interventions in clinical translation in the future.
